# The impact of exosomes derived from distinct sources on rheumatoid arthritis

**DOI:** 10.3389/fimmu.2023.1240747

**Published:** 2023-07-27

**Authors:** Sicheng Zhang, Zhen Duan, Fang Liu, Qingjie Wu, Xiwei Sun, Hailong Ma

**Affiliations:** ^1^ Department of Pediatric Orthopedics, Anhui Provincial Children’s Hospital, Hefei, China; ^2^ The Function Examination Center, Anhui Chest Hospital, Hefei, China

**Keywords:** exosome, stem cell, rheumatoid arthritis, inflammation, synovium

## Abstract

Rheumatoid arthritis (RA) is an autoimmune disease that can induce joint deformities and functional impairment, significantly impacting the overall well-being of individuals. Exosomes, which are cellularly secreted vesicles, possess favorable biological traits such as biocompatibility, stability, and minimal toxicity. Additionally, they contain nucleic acids, lipids, proteins, amino acids, and metabolites, serving as mediators in cellular communication and information exchange. Recent studies have demonstrated the association between exosomes and the pathogenesis of RA. Exosomes derived from mesenchymal stem cells, dendritic cells, and neutrophils exert influence on the biological functions of immune cells and joint cells, however, the precise mechanism remains largely unclarified. This comprehensive review systematically analyzes and summarizes the biological characteristics and functionalities of exosomes derived from diverse cellular sources, thus establishing a scientific foundation for the utilization of exosomes as diagnostic targets and therapeutic modalities in the context of RA.

## 1 Introduction

RA is a long-lasting and widespread inflammatory autoimmune disease that is characterized by inflammation in the synovial membrane, gradual erosion of the joints, and involvement of other body parts outside the joints (1). The prevalence rate of RA in China ranges from 0.2% to 0.4%, while European and American countries experience a higher prevalence rate of up to 1%. The pathogenesis of RA remains incompletely understood, and this immune disease is associated with a high disability rate, poor prognosis, and susceptibility to recurrent attacks (2). Additionally, RA can impact the synovial joint lining, causing stiffness, pain, inflammation, limited mobility, and joint erosion (3). Furthermore, the articular cartilage primarily comprises an extracellular matrix (ECM) and a small population of cells. The ECM mainly consists of type II collagen, proteoglycans, and aggrecans. In the context of RA, the synovium undergoes hyperplastic transformation into an invasive tissue that destructs cartilage and bone. Fibroblastoid synovial cells (FLSs), which line the joints, exhibit an aggressive phenotype in RA and play a crucial role in these pathological processes (4). FLSs, along with matrix metalloproteinases (MMPs) secreted by chondrocytes, constitute the key components contributing to cartilage tissue destruction (5). Abnormal proliferation of FLSs results in elevated levels of interleukin (IL)-6, IL-8 and other cytokines and chemokines, promoting the activation and migration of leukocytes from blood vessels to the synovium (6).

Exosomes are nanovesicles that originate from endosomes and possess a diameter ranging from 40 to 160 nm (with an average of 100 nm). These small vesicles are enclosed by lipid layers and can be released by various cells, and they are detectable in both tissues and biological fluids (7–10). Depending on their cellular origin, exosomes harbor diverse constituents, including DNA, RNA, lipids, metabolites, cytosolic proteins, and cell surface proteins (11, 12). Due to their ability to carry genetic information, exosomes serve as crucial mediators in intercellular communication and have been investigated as potential carriers for therapeutic molecules (13). Numerous studies have implicated exosomes in inflammatory processes, which play fundamental roles in the pathogenesis of numerous diseases such as cancer, type II diabetes, inflammatory bowel diseases, RA, and neurodegenerative diseases. Furthermore, exosomes have emerged as vital regulators of intercellular communication, exerting their influence locally and systemically by modulating a wide array of biological processes between cells. Notably, exosomes represent a cutting-edge treatment strategy for systemic immune diseases (14–16). Thus, aside from their involvement in the pathogenesis of RA, exosomes also exert significant influence in inflammation, cell signaling, immune regulation, and can potentially serve as biomarkers for diagnosing RA.

In this review, we conduct a comprehensive analysis and synthesis of the functions of various exosomes derived from cells in the pathogenesis of RA, as well as their potential preventive roles. Our findings offer valuable clinical insights into the potential diagnostic and therapeutic applications of exosomes as a means to identify future therapeutic targets for RA.

## 2 Exosomes derived from mesenchymal stem cells

Mesenchymal stem cells (MSCs) are a distinct population of cells characterized by their ability to undergo self-renewal and differentiate into multiple cell types. These cells possess the capacity to modulate inflammatory responses and play a crucial role in various pathological conditions associated with tissue repair and regeneration. MSCs can be obtained from diverse sources such as bone marrow, umbilical cord, adipose tissue, and other tissues (17). MSC therapies have been employed as cell-based therapeutic interventions for several decades due to their anti-inflammatory, immunomodulatory, and regenerative attributes (18). Several studies have suggested that exosomes released by MSCs (MSCs-Exo) not only demonstrate enhanced efficacy compared to the parent cells but also exhibit reduced toxicity and improved stability. These exosomes are capable of transferring various nucleic acids, proteins, and lipids from the donor cell to the recipient cell, thereby contributing to chronic inflammatory and immune processes (19, 20).

Exosomes derived from MSC can affect the occurrence and progression of RA through lncRNA, miRNA and circRNA. Su et al. discovered that exosomes derived from MSCs play a role in intercellular transfer of lncRNA HAND2-AS1, which leads to the suppression of RA-FLS activation through the miR-143-3p/TNFAIP3/NF-κB pathway. This finding provides a novel understanding of the pathogenesis and treatment of RA (21). Additionally, L. Chang et al. observed decreased levels of circFBXW7 and histone deacetylase 4 (HDAC4), along with elevated levels of miR-216a-3p in clinical RA samples compared to healthy samples. HDAC4 is involved in modulating immunity, inflammation, and osteoblast differentiation during the onset of RA, and it also contributes to the release of RA-related inflammatory cytokines by FLSs. L. Chang et al. demonstrated that treatment with exosomal circFBXW7 suppressed proliferation, migration, and inflammatory response of RA-FLSs, as well as attenuated damage in the RA model. The circFBXW7 directly acts as a sponge for miR-216a-3p, leading to the upregulation of HDAC4 expression. The therapeutic effects of exosomal circFBXW7 were diminished when HDAC4 was inhibited or miR-216a-3p was upregulated (22). Furthermore, H.Y. Meng et al. produced exosomes from human MSCs overexpressing miRNA-124a. They observed that co-incubation with HMSC-124a-EV effectively suppressed cell proliferation, migration, and promoted apoptosis in a fibroblast-like synoviocyte cell line. Their findings suggest that MSC-derived exosomes serve as efficient carriers for therapeutic miRNA, offering a promising avenue for developing new medicines and strategies to treat RA (23). Here, we summarize the role of different MSC derived exosomes in RA and the related mechanisms.

### 2.1 Exosomes derived from bone marrow mesenchymal stem cells

At present, the research on exosome in MSC s mainly focuses on bone marrow mesenchymal stem cells (BMSCs) (24–26). Fibrinogen-like protein 1 (FGL1) is a member of the fibrinogen family and can be recognized as an immune checkpoint target through an immune escape mechanism (27). FGL1 functions as an anti-inflammatory agent in collagen-induced RA (28). Subsequent investigations have demonstrated that FGL1 contained in MSCs-Exo exhibits therapeutic effects on RA without significant adverse reactions. Overexpression of FGL1 reduces the activity of the nuclear factor kappa B (NF-κB) pathway, thereby attenuating RA injury by inhibiting apoptosis of fibroblast-like synoviocytes (FLS) and promoting their proliferation (29). It is acknowledged that MMPs are involved in the degradation of the extracellular matrix (ECM). FLS can produce MMPs, among other matrix-degrading enzymes, which contribute to the destruction of cartilage in the affected joints of RA (30). Vascular endothelial growth factor (VEGF) is a potent growth factor specific to endothelial cells and is upregulated by pro-inflammatory cytokines and hypoxia. Serum concentrations of VEGF are elevated in RA and associate with disease activity (31). In a study by Chen et al., MSCs were transfected with an miR-150-5p expression plasmid, and MSC-derived exosomes were harvested. MSC-derived exosomes containing miR-150-5p may play a beneficial role in ameliorating joint destruction in RA. *In vivo* experiments demonstrated that MSC-derived miR-150-5p exosomes inhibit synoviocyte hyperplasia and angiogenesis by reducing the migration and invasion of FLSs and downregulating tube formation in human umbilical vein endothelial cells (HUVECs) through the targeting of MMP14 and VEGF. Injection of MSC-derived miR-150-5p exosomes leads to a reduction in hind paw thickness and clinical arthritic scores in a mouse model of collagen-induced arthritis, thus facilitating the direct intracellular transfer of miRNAs between cells and representing a potential therapeutic strategy for RA (13). Moreover, BMSCs have emerged as a viable solution for treating inflammatory rheumatism, they also have the potential to promote inflammation. BMSCs exhibit low immunogenicity and possess immunomodulatory effects, enabling them to regulate various cell types through the transmission of exosomes. These exosomes derived from BMSCs carry specific regulatory molecules present in the parent cells, including programmed death (PD)-L1, galectin-1 (GAL-1), and transforming growth factor (TGF)-β1 (32). In a murine model of collagen-induced arthritis (CIA), which mimics human RA, PD-L1 demonstrates the capacity to modulate collagen type II (CII)-reactive T cells and subsequently mitigate joint destruction. Intraperitoneal administration of PD-L1-Ig in mice leads to a deceleration in the development rate of CIA and an improvement in associated clinical manifestations (33). GAL-1 exerts regulatory functions within the immune system, with a study verifying increased levels of GAL-1 serum (sGal1) in RA patients (34).

Additionally, Stella et al. conducted the initial investigation on the involvement of exosomes derived from bone marrow mesenchymal stem cells (BMSC-Exo) in models of RA. They demonstrated the effective therapeutic potential of BMSC-Exo in mitigating experimental RA. This was achieved by dose-dependent inhibition of T lymphocyte proliferation and reduction in the proportion of CD4+ and CD8+ T cell subsets. Notably, the administration of parental MSCs did not result in an increase in Treg cell population. In a study using delayed-type hypersensitivity (DTH) mice, a dose-dependent anti-inflammatory effect of BMSC-Exo was observed. Furthermore, BMSC-Exo effectively alleviated clinical symptoms of inflammation in a mouse model of collagen-induced arthritis (CIA). The beneficial impact of BMSC-Exo was correlated with a decrease in plasmablast numbers and an increase in Breg-like cell numbers in lymph nodes (26). Another study indicated that exosomes derived from BMSCs have the ability to hinder the release of IL-1β, tumor necrosis factor-α (TNF-α), and IL-18, as well as the activation of NLRP3 in macrophages and RA rats. Additionally, they confirmed that BMSC-derived exosomes containing miR-223 could ameliorate RA by inhibiting NLRP3 expression in macrophages (35). G.Q. Li et al. substantiated that BMSC-derived exosomes alleviate RA by delivering miR-21. The exosomal miR-21, in turn, alleviates RA by targeting the TET1/KLF4 regulatory axis. TET1, a member of the DNA demethylase family that governs the expression of numerous genes, has been associated with RA. It is noteworthy that KLF4, a key player in cell survival and proliferation, has been recently found to be upregulated in RA (36).

### 2.2 Exosomes from human umbilical cord mesenchymal stem cells

The easier obtainability of umbilical cord mesenchymal stem cells (UMSCs) compared to other cell types is complemented by their ability to maintain their biological properties unchanged even after cryopreservation. UMSCs have progressively emerged as the preferred cells for cell therapy, gradually replacing bone marrow-derived MSCs. Human UMSCs (HUMSCs) release exosomes that exhibit specific immunomodulatory functions in the context of RA. Notably, macrophages, B cells, T cells, particularly CD4+ T cells, assume pivotal roles in local inflammation development. The immune pathogenesis of RA is linked to the imbalanced response of memory Th17 and memory regulatory T cells. The progression of RA is regulated by T helper and regulatory T (Treg) cells, with synovial inflammation and pannus growth being attributed to Th17 cells. A particular study highlighted the significant role of HUMSC-Exo in regulating the balance between Th1/Th17 and Treg cells during immune and inflammatory responses, thus reducing the ratio of Th1/Th17 to Treg cells and inhibiting the development of RA. Additionally, HUMSC-Exo may exert a direct influence on macrophage and osteoclast differentiation (37). In light of these findings, it can be inferred that exosomes derived from HUMSCs have the potential to modulate the equilibrium between pro-inflammatory and anti-inflammatory cells, presenting a promising therapeutic avenue for RA.

Serum/glucocorticoid regulated kinase 1 (SGK1) serves as a crucial modulator in the process of osteo-/chondrogenic transdifferentiation and calcification in vascular smooth muscle cells. Exosomal miRNA-140-3p derived from HUMSCs effectively mitigates joint injury in rats with RA by downregulating the expression of SGK1. Overexpression of SGK1 reversed the inhibition of RASF growth caused by overexpression of miR-140-3p (38).

### 2.3 Exosomes derived from synovial mesenchymal stem cells

Zhang J. et al. discovered that the intracellular transfer of circEDIL3 through exosomes derived from synovial mesenchymal stem cells (SMSC-Exo) holds potential as a novel therapeutic approach for RA. The circEDIL3 molecule functions as a sponge that specifically targets miR-485-3p, which in turn regulates PIAS3. PIAS3, a member of the small Rho GTPase family, serves as a primary cellular inhibitor of STAT3. By inhibiting STAT3 activity, PIAS3 exosomes derived from SMSCs overexpressing circEDIL3 effectively downregulated the expression of the VEGF complex. This effect was achieved by influencing the miR-485-3p target through PIAS3 and suppressing STAT3 activity, resulting in reduced downstream VEGF levels. These findings were observed in the supernatants of co-cultured RA-FLS (rheumatoid arthritis fibroblast-like synoviocytes) and human dermal microvascular endothelial cells (HDMECs), as well as in the cell lysate of co-cultured RA-FLSs (39). These studies have demonstrated that exosomes derived from MSCs effectively attenuate the invasion and migration of FLS to some extent.

**Figure 1:**
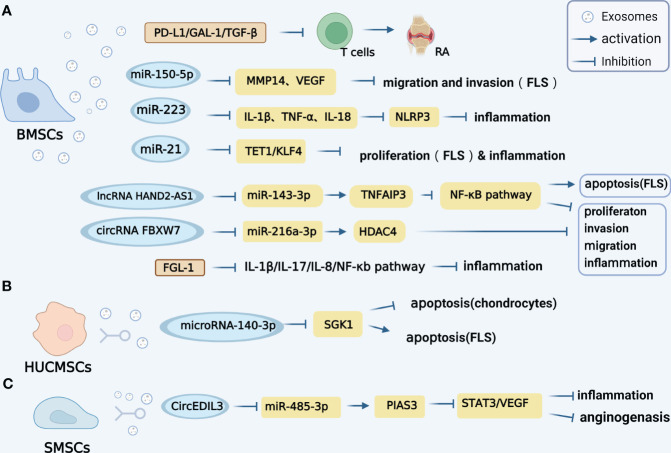
Mechanisms of diverse MSC-derived exosomes on RA. **(A)**. Exosomes derived from (BMSCs contain various regulatory molecules, such as PD-L1, GAL-1, and TGF-β1, which play a significant role in promoting the progression of RA. Additionally, BMSCs-derived exosomes carrying miR-150-5p have been found to effectively reduce the migration and invasion of RA-FLS cells by targeting VEGF and MMP14. Furthermore, the exosomal miR-223 derived from BMSCs exhibits inhibitory effects on the release of interleukin-1β (IL-1β), tumor necrosis factor-α (TNF-α), and interleukin-18 (IL-18), as well as the activation of NLRP3 in rats, thereby alleviating inflammation. Notably, BMSCs-derived exosomes also participate in the intercellular transfer of lncRNA HAND2-AS1. The lncRNA HAND2-AS1 has been shown to have a positive impact on RA by inhibiting miR-143-3p and promoting the expression of TNFAIP3, resulting in the inhibition of the NF-κB pathway. In terms of circRNA, the BMSCS-derived exosome CircFBXW7 has been found to directly inhibit the activity of miR-216a-3p and up-regulate the expression of HDAC4, leading to the inhibition of proliferation, migration, and inflammatory response in RA-FLS. Furthermore, FGL1-MSC-Exo has the ability to reduce inflammatory cytokines such as IL1-β, IL-17, IL-8, and the NF-κB pathway, thereby attenuating RA injury. **(B)**. HUMSCs-derived exosomes miRNA-140-3p can attenuate joint injury of rats with rheumatoid arthritis by silencing SGK1. **(C)**. SMSCs-derived exosomes circEDIL3 can act as a sponge targeting miR-485-3p of PIAS3, inhibit STAT3 activity and reduce downstream VEGF.

The specific mechanisms of diverse MSC-derived exosomes on RA are summarized in **Figure 1**. Nevertheless, there is a dearth of research investigating the relationship between SMSC-Exo and RA, a lot of research needs to be carried out in the future.

## 3 Exosomes derived from dendritic cells

Dendritic cells (DCs) play a crucial role in initiating antigen-specific immune responses and promoting immune tolerance (40, 41). Recently, there has been growing interest in exploring and utilizing exosomes derived from DCs in the context of autoimmune diseases. A study demonstrated that exosomes derived from immature DCs exhibited therapeutic potential in treating mice with collagen-induced arthritis (CIA). These exosomes, when derived from immature DCs treated with IL-10, effectively suppressed inflammation and autoimmune responses by inhibiting pro-inflammatory cytokines such as IL-1 and TNF-α, as well as reducing Hsp70 levels. However, it was observed that DC-derived exosomes (DC-Exo) showed diminished responsiveness to regulatory T cells compared to DCs alone (42). Subsequent investigations revealed that exosomes obtained from DCs overexpressing indoleamine 2,3-dioxygenase (IDO) or CTLA-4Ig, an inducer of IDO, could reverse established CIA and alleviate inflammation in a model of delayed-type hypersensitivity (DTH) in mice. The DC-derived exosomes were found to suppress CD8+ effector T cells and interact with endogenous antigen-presenting cells through B7 costimulatory molecule-dependent mechanisms, thereby modulating their function (43). IDO, an immunomodulatory protein known for its role in inducing or maintaining peripheral tolerance and immunosuppression in autoimmune diseases, asthma, cancer, is upregulated by CTLA-4Ig and has shown promise in the treatment of RA (44, 45). Furthermore, exosomes secreted by genetically modified bone marrow-derived DCs were found to secrete IL-4 and exhibited therapeutic effects by reducing the severity and incidence of CIA, as well as inhibiting inflammation in DTH mice. These DC-derived exosomes exerted their inhibitory effects on the DTH response through MHC class II molecules, partially dependent on Fas ligands/Fas, thereby modulating the activity of collagen-reactive T cells in *in vivo* (46). Current research suggests that DC-derived exosomes not only possess immunosuppressive properties but also hold potential as carriers for drug delivery in immune-related disorders. Triptolide, a compound known for its immunosuppressive effects, selectively targets DCs. *In vivo* experiments demonstrated that DC-derived exosomes can encapsulate triptolide, enabling targeted delivery of the compound and alleviating local inflammation and damage in mice with RA, while reducing toxicity. Additionally, triptolide-loaded DC-derived exosomes were found to reshape the immune microenvironment by reducing the levels of CD4+ T cells and increasing the levels of Treg cells in the body (47). Therefore, DC-derived exosomes can serve as carriers for anti-RA drugs and offer a novel non-cellular drug delivery system, presenting a promising approach for anti-RA therapy. Nevertheless, there is a paucity of research on the specific molecules present in exosomes derived from dendritic cells, which display therapeutic effects on RA. This deficiency requires prompt attention, and further investigations are imperative.

## 4 Exosomes derived from neutrophils

Neutrophils are abundant in the synovial fluid of patients with RA. A study has identified an increased concentration of polymorphonuclear neutrophil-derived exosomes (PMN-Exo) in the synovial fluid of RA patients. PMN-Exo induces an elevation of Annexin A1 (AnxA1+) in the synovial fluid, which activates anabolic genes in chondrocytes and contributes to extracellular matrix (ECM) accumulation and cartilage protection by regulating IL-8 and prostaglandin E2 (PGE2). Correspondingly, *in vivo* experiments have demonstrated that intra-articular injection of AnxA1 (+)-containing exosomes attenuated cartilage degeneration in mice with inflammatory arthritis. Furthermore, direct co-culture of neutrophils with chondrocytes indicated that chondrocyte death was induced, whereas exposure to neutrophil exosomes exerted a protective effect. It was revealed that exosomes, rather than neutrophils themselves, possess the ability to penetrate cartilage (48). Another study suggested that exosomes secreted by neutrophils, due to nanase functionalization, exhibit excellent anti-inflammatory properties. Ultrasmall Prussian Blue nanoparticle exosomes (UPB-Exo) selectively accumulate in activated FLS, subsequently neutralizing pro-inflammatory factors, eliminating reactive oxygen species, and alleviating inflammatory stress. Moreover, UPB-Exo can effectively target inflammatory synovitis and penetrate the cartilage, enabling precise diagnosis of RA *in vivo* and triggering a series of anti-inflammatory events through regulation of Th17/Treg cell balance, thus significantly improving joint injury (49).

## 5 Exosomes derived from granulocytic myeloid-derived suppressor cells and macrophages

**Figure 2:**
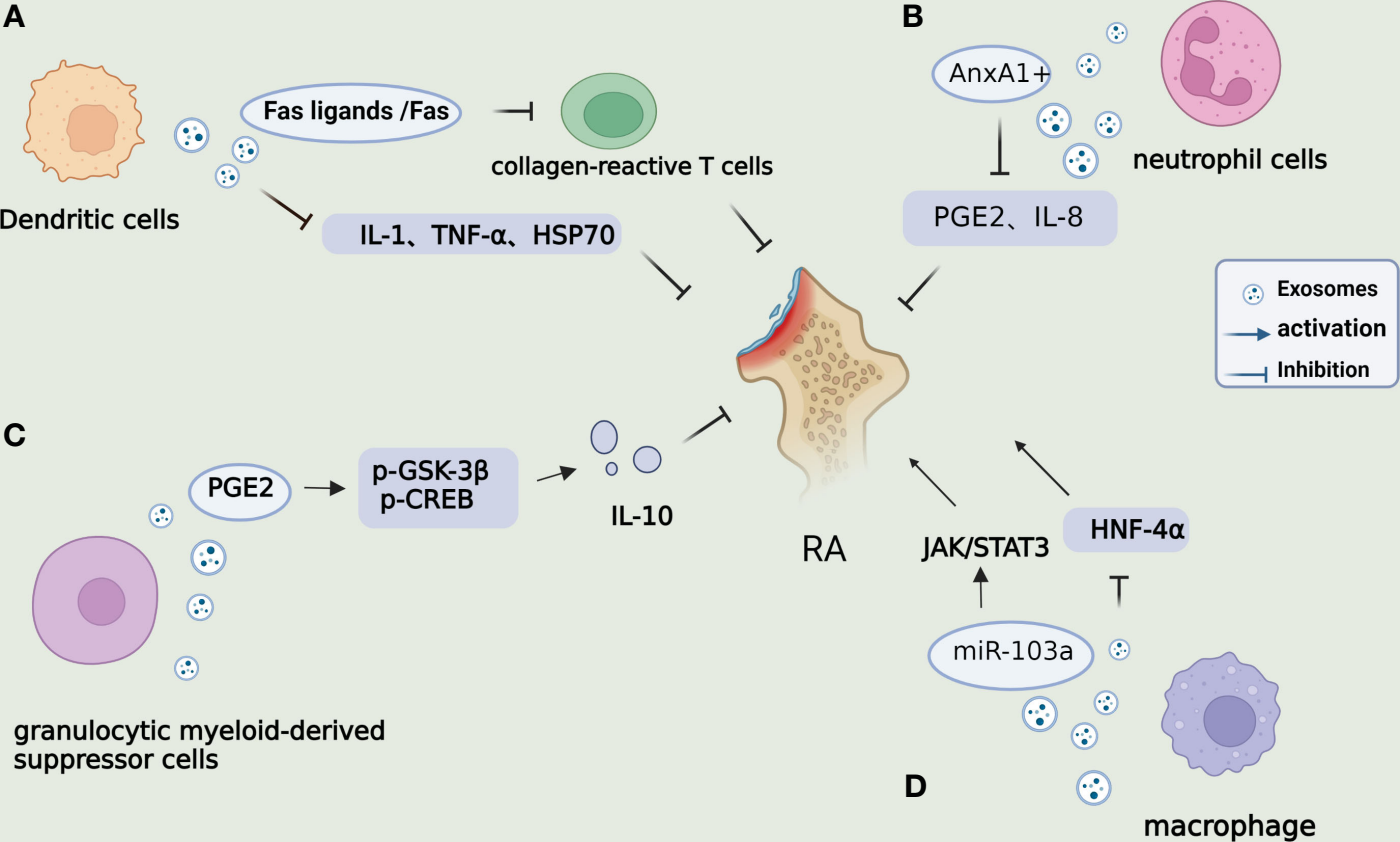
Mechanisms of other cell-derived exosomes on RA. **(A)**. DC-derived exosomes can inhibit the DTH mice response through MHC class II, partially dependent on Fas ligands/Fas, thereby regulating the activity of collagen-reactive T cells. DCs treated with IL-10 can inhibit inflammation and autoimmune responses by inhibiting pro-inflammatory cytokines IL-1 and TNF-α and reduce Hsp70 levels. **(B)**. Annexin A1 (AnxA1^+^) is overexpressed by PMN-derived exosomes in synovium fluid, which can activate anabolic genes in chondrocytes and play a role in ECM accumulation and cartilage protection by regulating IL-8 and PGE2. **(C)**. Exosomes derived from G-MDSC can produce PGE2 and upregulate phosphorylation of GSK-3β and CREB, promoting the secretion of IL-10 from regulatory B cells and ameliorating arthritis in mice. **(D)**. miR-103a in RAW264.7-derived exosomes from macrophages can promote inflammation and angiogenesis in RA mice by targeting the downregulation of HNF-4α and activation of the JAK/STAT3 pathway, thus, aggravating RA in mice.

GMDSC-Exo, secreted by granulocytic myeloid-derived suppressor cells, exhibits regulatory effects on immune cells. In both *in vivo and in vitro* settings, GMDSC-Exo stimulates the secretion of IL-10 by regulatory B cells. Arthritis patients and mice demonstrate elevated levels of PGE2 in their serum and synovium. Upon injection of GMDSC-Exo into CIA mice, a decrease in arthritic index values and inflammatory cell infiltration is observed. Previous research has shown that PGE2 can elevate the levels of IL-10, an anti-inflammatory cytokine, and is a crucial factor in the production of IL-10 and Breg cells. Studies have revealed that GMDSC exosomes can generate PGE2, upregulate the phosphorylation of GSK-3β and CREB, and exert an anti-inflammatory role by promoting the secretion of IL-10 (50). Macrophages play a significant role in complex microenvironments and can be categorized into M1 and M2 subtypes. Imbalances between pro-inflammatory M1 and anti-inflammatory M2 macrophage activities induce synovial inflammation and autoimmunity, leading to joint injury. It has been reported that macrophage-derived exosomes not only enhance inflammation and cellular immune responses but also serve as nanocarriers for drug delivery in therapeutic applications. Plasmid DNA encoding the anti-inflammatory cytokine IL-10 (IL-10 pDNA) and the chemotherapy drug betamethasone sodium phosphate (BSP) can be encapsulated within exosomes (M2-Exo) derived from M2-type macrophages. This co-delivery system of M2 Exo/pDNA/BSP promotes the polarization of M1-to-M2 macrophages by reducing the secretion of pro-inflammatory cytokines (IL-1β and TNF-α) while increasing the expression of IL-10. *In vivo* experiments also demonstrate the potent anti-inflammatory activity of M2 Exo/pDNA/BSP. Furthermore, M. Chen et al. have discovered that high expression of miR-103a in exosomes derived from macrophages (RAW264.7) can exacerbate inflammation and angiogenesis in RA mice by downregulating hepatocyte nuclear factor 4 alpha (HNF-4α) and activating the JAK/STAT3 pathway, thereby aggravating RA in mice (51). These studies present empirical evidence indicating that exosomes originating from GMSCs and macrophages may mitigate the advancement of RA by means of their anti-inflammatory properties. In brief, **Figure 2** illustrates the mechanisms of exosomes derived from DCs, neutrophils, GMDSCs, and macrophages within the framework of RA. Additional research is necessary to elucidate the exact molecular mechanisms that underlie exosome-mediated signaling pathways in various cell types in the future.

## 6 Exosomes derived from serum and plasma

In recent years, there has been a growing body of research focusing on the role of exosomes in arthritis serum. Several studies have been conducted by numerous researchers, who have extracted serum exosomes from both clinical RA patients and collagen-induced arthritis (CIA) mouse models. These investigations aimed to analyze the influence of serum exosomes on RA morbidity, clinical score, and bone degradation. The results of these studies suggest a noteworthy association between serum exosomes and the occurrence of RA. As a result, the timely identification and prevention of RA are of utmost importance. However, it remains unclear whether serum exosomes accurately reflect the content of exosomes in synovial fluid. Exosomes were isolated from the serum and synovial fluid of osteoarthritis patients, and miRNA expression was compared through miRNA sequencing. The comparison revealed 31 upregulated miRNAs and 33 downregulated miRNAs in the synovial fluid compared to the serum. Further transcription analysis demonstrated that these differentially expressed miRNAs primarily relate to intercellular processes and metabolic pathways. Hence, the results suggest that serum-derived exosomes do not fully represent synovial exosomes (52). In another study utilizing global miRNA screening in plasma exosomes, performed with a custom microarray on both RA patients and healthy controls, researchers identified four abnormally expressed exosomal miRNAs in RA patients. The downregulation of exosomal miR-204-5p was confirmed in both the replication group (30 RA patients vs. 30 healthy controls) and the validation group (56 RA patients vs. 60 healthy controls). Furthermore, Spearman correlation analysis indicated an inverse correlation between the expression of plasma exosomal miR-204-5p and disease parameters of RA patients, including rheumatoid factor (RF), erythrocyte sedimentation rate (ESR), and C-reactive protein (CRP) levels (53). Another study demonstrated that overexpression of serum-derived exosomes containing nuclear-enriched abundant transcript 1 (NEAT1) or ROCK2 promotes the proliferation of immune cells (CD4+ T cells), differentiation of Th17 cells, and cell migration in response to stimulation. NEAT1 binds to and inhibits the expression of miR-144-3p, and knockout of the NEAT1 gene induces the expression of miR-144-3p in CD4+ T cells. MiR-144-3p is associated with the activation of ROCK2 in RA, thereby activating the Wnt/β-catenin pathway (54). Moreover, a novel group of serum biomarkers consisting of exomiR-451a, exomiR-25-3p, and serum sTWEAK levels may be utilized for early clinical diagnosis of RA. Additionally, a newly identified predictive RNA target gene, YHWAB, may play a significant role in the development of RA (55). Osteoclasts directly transform into osteoblasts, and elevated levels of miR-214-3p in osteoclasts are associated with increased serum exosome miR-214-3p and reduced bone formation in older women with fractures and ovariectomized (OVX) mice. Targeted inhibition of miR-214-3p promotes bone formation in aging OVX mice (56). Furthermore, exosome-encapsulated miR-6089 exhibits a significant reduction in serum exosomes in 76 RA patients compared to 20 controls. MiR-6089 regulates the production of IL-6, IL-29, and TNF-α by targeting TLR4 signal transduction, thereby inhibiting cell proliferation induced by lipopolysaccharide (LPS) and macrophage-like activation of THP-1 cells. Therefore, exosome-encapsulated miR-6089 holds promise as a new biomarker for RA (57). Moreover, the expression of miR-3a-76p in serum exosomes and peripheral blood mononuclear cells (PBMCs) of RA patients (n = 20) was significantly downregulated compared to healthy controls (n = 548). Serum exosome miR-548a-3p exhibited a negative correlation with serum CRP, RF, and ESR levels in RA patients.

Toll-like receptors (TLRs) are transmembrane proteins that recognize diverse pathogen-associated molecular patterns, triggering an inflammatory immune response. All TLRs engage the myD88-dependent pathway, which activates NF-κB, a classical inflammatory pathway, and ultimately elicits the release of inflammatory mediators and cytokines. Wang et al. previously identified the distinct exosome-encapsulated miRNA profile in serum samples of RA patients using miRNA microarray analysis. This profile encompasses 20 differentially expressed serum exosomal miRNAs. Notably, miR-548a-3p stands out as one of the significantly down-regulated exosome-delivered miRNAs in the serum of RA patients when compared to healthy controls. Subsequently, they determined that miR-548a-3p exerts inhibitory effects on the proliferation and activation of pTHP-4 cells by modulating the TLR1/NF-κB signaling pathway (58). Furthermore, the expression level of HOX antisense intergenic RNA (Hotair) in serum exosomes of patients with RA exhibited a substantial increase, leading to the migration of activated macrophages. Conversely, inhibition of Hotair resulted in the reduction of MMP-2 and MMP-13 levels. These findings indicate that, in addition to miRNAs, lncRNAs transcribed in reverse orientation may also contribute to the pathogenesis of RA through serum-derived exosomes (59).

## 7 Discussion

In conclusion, RA is a systemic and progressive inflammatory disorder that results in joint and periarticular structural damage as well as systemic inflammation-related consequences. Exosomes, which are small nanovesicles released by nearly all cells, contain genetic information and have emerged as crucial mediators of intercellular communication in various biological processes. Through the investigation and analysis of the presence of miRNAs and lncRNAs in exosomes derived from different cell types and serum sources, it has been observed that exosomes predominantly contribute to immune regulation, control of inflammatory response, and reduction of inflammatory cytokine release. Exosomes actively participate in the pathogenesis of RA, offering potential therapeutic prospects in inflammation management, cellular signaling, and immune regulation. Furthermore, exosomes exhibit great potential as biomarkers for RA diagnosis and as a diagnostic tool for precise identification and targeted treatment. Among these, MSC-Exo demonstrates significant potential in the treatment of RA. Neutrophils, granulocytic myeloid-derived suppressor cells, and macrophage-secreted exosomes also exert immunological regulatory and anti-inflammatory roles in RA. The miRNAs and lncRNAs enclosed within exosomes derived from serum could serve as novel groups of serum biomarkers for early clinical detection of RA. Additionally, certain exosomes derived from stem cells act as therapeutic agents in RA by functioning as drug carriers. Consequently, scrutinizing the roles of exosomes derived from different sources in the pathogenesis of RA and their preventive functions holds promise for the identification of future therapeutic targets.

## Author contributions

SZ designed and wrote this manuscript, ZD wrote and checked this manuscript, FL wrote thismanuscript, QW checked the manuscript, XS checked themanuscript, HM designed and checked the manuscript. Allauthors contributed to the article and submitted and approvedthe submitted section.
